# Examination of histopathological growth patterns of liver metastases in a retrospective, consecutive, single-center, cohort study

**DOI:** 10.3389/pore.2025.1612161

**Published:** 2025-10-30

**Authors:** Anita Sejben, Parsa Abbasi, Boglárka Pósfai, Tamás Lantos

**Affiliations:** ^1^ Department of Pathology, University of Szeged, Szeged, Hungary; ^2^ Department of Medical Physics and Informatics, Albert Szent-Györgyi Medical School, University of Szeged, Szeged, Hungary

**Keywords:** liver metastasis, histopathological growth pattern, single-center study, secondary liver cancer, grade

## Abstract

**Objective:**

Histopathological growth patterns (HGPs) were identified as prognostic factors for colorectal adenocarcinomas; however, they have been examined in a consecutive setting with controversial results. Our study aimed to examine HGPs’ association with clinicopathological factors in a retrospective, consecutive, single-center, cohort study.

**Methods:**

Our study comprised the data of patients who were treated for liver metastases from 2011 to 2023. In all cases, general clinicopathological data were registered. The histological slides of all metastatic foci were individually evaluated. Statistical analyses were carried out by using the Kruskal-Wallis and Fisher’s exact test. P-values less than 0.05 were considered significant.

**Results:**

Altogether 336 liver metastases from 205 patients have been included in our retrospective, consecutive, single-center, cohort study. The male-to-female ratio was 116:89, and the average age of patients was 68 years (median: 69.5; range: 27-93). Most examined cases were of colorectal origin (n = 164). Replacement pattern was found to be the most common (n = 99). The 163 colorectal adenocarcinoma metastasis cases reflected a similar order of magnitude of replacement type (n = 78) and desmoplastic (n = 68) HGPs. The majority (70%) of neuroendocrine tumours (n = 10) showed pushing HGP, while 3 of 5 non-epithelial tumours were associated with replacement-type HGP. A significant association was found between HGPs and histological subtype (*p < 0.001*), grade (*p = 0.002*), the presence of venous spread (*p = 0.02*), and the largest diameter of liver metastasis (*p = 0.023*).

**Conclusion:**

Even though our study highlights the HGPs’ association with several clinicopathological parameters that might influence prognosis, their role in the treatment process of colorectal or other carcinomas remains controversial.

## Introduction

Distant metastases are still widespread causes of death in cancer patients [1]. It has been evident for a while now that primary and secondary tumours do not necessarily behave the same way; therefore, the examination of metastases has become more important [2]. The liver serves as a frequent target of metastases, due to its anatomic connection to the portal vein system; hence, gastrointestinal, pulmonary, mammary cancers, and other tumours, such as melanomas, often involve it [3, 4]. Liver metastases may show a wide range of clinical behaviour as well, due to the different primary tumour biology and the interaction between the metastatic cells and the liver microenvironment [5].

Histopathological growth patterns (HGPs) have been earlier identified primarily as a prognostic factor in colorectal cancer, however, several studies revealed their utility in consecutive settings, regardless of histological characteristics, as well, however, with controversial results [2, 3, 5–9]. According to the study by Meyer et al, which analysed different kinds of carcinoma and melanoma cases, and soft tissue tumours, a significant association was found between HGPs and overall survival (OS), and recurrence-free survival (RFS) [9]. On the other hand, Bohlok and coworkers’ work with a similar diagnostic palette reflected an association with post-operative overall survival (POOS) and progression-free survival (PFS) [5]. The consecutive manner raises theoretical concerns, since the biology, and therefore, the behaviour of epithelial, melanocytic, and mesenchymal tumours are completely different. Furthermore, the predictive value of HGPs has been described by van Dam et al, while according to their review, HGPs may forecast colorectal cancer’s therapeutic response to bevacizumab [10].

Even though HGPs can be easily assessed, since only hematoxylin and eosin (HE) stained slides and an optical microscope are sufficient to determine the liver-metastasis interface, and they have been the focus of attention by several study groups in the last 40 years, their reproducibility and prognostic value in a consecutive setting has not been finalised, therefore, they are still not included worldwide in the routine histopathological reports, and they do not currently influence therapeutic decisions [11–13].

Our main objective for this study was to examine HGPs in a retrospective, consecutive, single-center cohort over a period of 13 years in a university center that has a gastrointestinal profile, and to determine their association with clinicopathological factors.

## Materials and methods

### Patient selection and eligibility criteria

Our study was designed in a retrospective, consecutive, cohort design, in a university institution that serves as a gastrointestinal center for the Southern Hungarian region (University of Szeged, Szeged, Hungary). Our database comprised patients with surgical specimens due to liver metastases (C7870) from 2011 to 2023. In our database, age, gender, histological subtype, date of primary tumour diagnosis, largest macroscopic diameter, clinical stage, TNM, grade, presence of venous spread, completeness of resection, and therapy of primary tumour were obtained from medical charts. Concerning the metastases, the date of diagnosis, intrahepatic localisation, type of surgery, focality, and largest macroscopic diameter were attained.

Patients, who were treated with chemotherapy less than 6 months before the liver metastasis surgery, were excluded from our study, while preoperative systemic chemotherapy has been known to alter HGPs [14]. Furthermore, only cases with at least 2 representative HE slides and paraffin-embedded blocks available were examined. Cases that did not contain tumour-free liver tissue were excluded. Subcapsular metastatic cases were eliminated, since according to the examiners’ earlier observations, these cases tend to resemble the desmoplastic pattern. If necessary, new, deeper HE sections were requested. While all tumour foci were separately evaluated, those cases that had differing HGPs due to multifocality were excluded, as well.

This study was approved by both the Regional and Institutional Research Ethics Committee of the University of Szeged (5462; 170/2023-SZTE) and the Medical Research Council (BM/5299-2/2024).

### Evaluation of HGPs

For our study, the HE slides were acquired from the archives of the Department of Pathology, University of Szeged. Metastatic foci were individually examined, and their HGP category was independently registered. During the evaluation process, a 3-headed consultation microscope was used (Olympus BX53; PA, BP, and AS), and the diagnosis of the HGP subtype was finalised by a fellowship-trained gastrointestinal pathologist, with 3 years of experience (AS). A short training session with a discussion of the main features of the HGP subtypes was held. The evaluation was carried out according to the guidelines of Latacz et al [13]. Desmoplastic HGP was defined by angiogenesis, and if the tumour was surrounded by a fibrous band, separating it from the non-tumourous liver parenchyma. In the replacement type, the cancer cells had to show continuity with the hepatocytes, while in pushing HGP, the expansile spread of the tumour was observed, with clear distinction. In the sinusoidal spread, the cancer cells were proliferating either in the sinusoids or in the Disse spaces. Portal spread was defined as tumour growth in the portal tracts, septa or biliary branches. In each case, solely a dominant pattern was identified that occupied at least 51% of the case [13, 15].

### Statistical analysis

Statistical analyses were carried out by the R statistical software (v4.1.1). To compare more than 2 independent groups with non-normally distributed data, the Kruskal-Wallis test was used, followed by the Dwass-Steel-Critchlow-Fligner (DSCF) test for *post hoc* pairwise comparisons with p-value adjustment for multiple testing. The association between categorical variables was examined using Fisher’s exact test, with p-values adjusted for multiple comparisons using the Holm-Bonferroni correction. All statistical tests were two-sided, and p-values less than 0.05 were considered statistically significant.

## Results

### General clinicopathological data and primary tumour characteristics

Altogether 336 liver metastases from 205 patients have been included in our retrospective, consecutive, single-center, cohort study. The male-to-female ratio was 116:89, and the average age of patients was 68 years (median: 69.5; range: 27–93). The median largest diameter of the primary tumour was 31 mm (range: 4–142 mm), while the median largest diameter of the liver metastasis proved to be 29 mm (range: 4–149 mm). The majority of tumours proved to be clinical stage 3 (n = 85) and 2 (n = 46), and grade 2 (n = 179). In most cases (n = 128), the patients were given adjuvant chemotherapy. The investigated clinicopathological features are highlighted in Table 1.

**TABLE 1:** Clinicopathological parameters and results of statistical analysis.

Variables	All cases (n = 205)	Replacement HGP (=99)	Desmoplastic HGP (n = 77)	Pushing HGP (n = 29)	p values
**Mean age (years) [standard deviation]**	68.1 [10.1]	69.3 [9.1]	67.2 [10.6]	66.8 [11.4]	*0.284*
**Gender**					*0.898*
Male	116 (57%)	54 (54.5%)	45 (34.7%)	16 (55.2%)	
Female	89 (43%)	45 (44.5%)	32 (65.3%)	12 (44.8%)	
**Main histological subclassification of primary tumours**	205 (100%)				***<0.001***
Colorectal	163 (79.5%)	78 (78.8%)	68 (88.3%)	17 (58.7%)	
Other epithelial, non-neuroendocrine	27 (13.2%)	17 (17.2%)	6 (7.8%)	4 (13.8%)	
Neuroendocrine	10 (4.9%)	1 (1%)	2 (2.6%)	7 (24.1%)	
Non-epithelial	5 (2.4%)	3 (3%)	1 (1.3%)	1 (3.4%)	
**Median of largest diameter of primary tumour (mm) [range]**	31 [4–142]	31.5 [5–95]	32 [5–95]	30 [4–142]	*0.845*
**Clinical stage at the time of primary tumour diagnosis**					*0.836*
Stage 1	43 (21%)	20 (20.2%)	14 (18.1%)	9 (31.1%)	
Stage 2	46 (22%)	26 (26.2%)	15 (19.5%)	5 (17.2%)	
Stage 3	85 (42%)	39 (39.4%)	36 (46.8%)	10 (34.5%)	
Stage 4	31 (15%)	14 (14.2%)	12 (15.6%)	5 (17.2%)	
Pathological stage at the time of primary tumour diagnosis					
T stage					*0.634*
T1	8 (3.9%)	2 (2%)	4 (5.2%)	2 (6.9%)	
T2	32 (15.6%)	14 (14.1%)	12 (15.6%)	6 (20.7%)	
T3	113 (55.1%)	55 (55.6%)	45 (58.4%)	13 (44.8%)	
T4	52 (25.4%)	28 (28.3%)	16 (20.8%)	8 (27.6%)	
N stage					*0.087*
N0	88 (42.9%)	48 (48.5%)	27 (35.1%)	13 (44.8%)	
N1	82 (40%)	35 (35.4%)	39 (50.6%)	8 (27.6%)	
N2	35 (17.1%)	16 (16.1%)	11 (14.3%)	8 (27.6%)	
M stage					*0.601*
M0	167 (81.5%)	83 (83.8%)	61 (79.2%)	23 (79.3%)	
M1	38 (18.5%)	16 (16.2%)	16 (20.8)	6 (20.7%)	
**Grade of primary tumour**					***0.002***
Grade 1	14 (7%)	4 (4%)	3 (3.9%)	7 (24.1%)	
Grade 2	179 (87%)	87 (88%)	72 (93.5%)	20 (69%)	
Grade 3	12 (6%)	8 (8%)	2 (2.6%)	2 (6.9%)	
**Presence of venous spread in primary tumour specimen**					***0.02***
Present	35 (17%)	19 (19%)	7 (9%)	9 (31%)	
Not present	170 (83%)	80 (81%)	70 (91%)	20 (69%)	
**Resection of primary tumour**					*0.933*
Complete	143 (69.8%)	74 (74.7%)	67 (87%)	23 (79.3%)	
Incomplete	62 (30.2%)	25 (25.3%)	10 (13%)	3 (20.7%)	
**Focality of metastasis**					*0.967*
Unifocal	112 (54.6%)	54 (54.5%)	41 (53.2%)	16 (65.5%)	
Multifocal	93 (45.4%)	45 (44.5%)	32 (46.8%)	13 (34.5%)	
**Median of largest diameter of liver metastasis (mm) [range] (n = 336)**	29 [4–149]	35 [6–135]	24.5 [4–115]	41 [6–149]	***0.023***

Bold p values indicate statistical significance (p<0.05).

### Examination of metastases and assessment of HGPs

**FIGURE 1:**
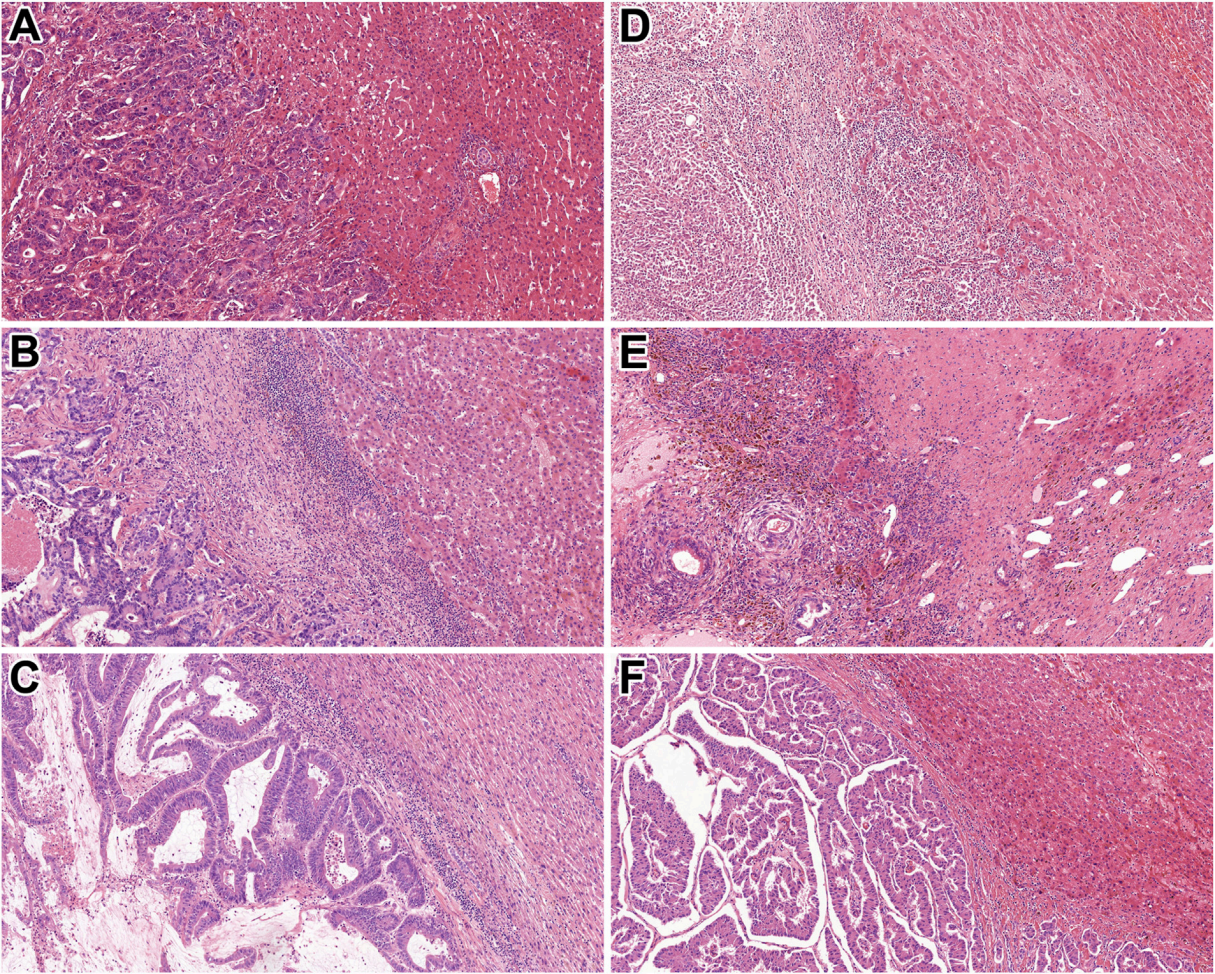
Histological characteristics of the identified HGPs. **(A)** Rectal adenocarcinoma metastasis with replacement HGP (HE, 10x). **(B)** Colonic adenocarcinoma metastasis displaying desmoplastic HGP (HE, 10x). **(C)** Rectal adenocarcinoma metastasis with pushing HGP (HE, 10x). **(D)** Malignant melanoma showing replacement HGP (HE, 10x). **(E)** Mixed germ cell tumour exhibiting desmoplastic HGP (HE, 10x). **(F)** Pancreatic NET demonstrating pushing HGP (HE, 10x). Abbreviations: HE - Hematoxylin and eosin, HGP - Histopathological growth pattern, NET – Neuroendocrine tumour.

Replacement pattern was found to be the most common (n = 99). Sinusoidal or portal HGPs were not identified at all in our cohort. The 163 colorectal adenocarcinoma metastasis cases reflected a similar order of magnitude of replacement type (n = 78) and desmoplastic (n = 68) HGPs. The majority of (70%) of neuroendocrine tumours (n = 10) were evaluated to show pushing HGP, while 3 of 5 non-epithelial tumours were associated with replacement-type HGP. Figure 1 represents the replacement, desmoplastic, and pushing HGPs in epithelial and non-epithelial tumours. During the statistical analysis, the histological subtype of primary tumours was classified into colorectal, other types of epithelial tumours, non-neuroendocrine, neuroendocrine, and non-epithelial.

The “global” Fisher’s exact tests indicated significant associations between HGP and histological subtype (*p < 0.001*), tumour grade (*p = 0.002*), and the presence of venous spread (*p = 0.02*), respectively. Post-hoc Fisher’s exact tests were conducted for all pairwise HGP comparisons. Holm-Bonferroni-adjusted p-values revealed that pushing HGP differed significantly from both replacement HGP and desmoplastic HGP in the case of the histological subtype (*p = 0.001* and *p = 0.002*, respectively) and tumour grade (*p = 0.008* and *p = 0.008*, respectively) and only from desmoplastic HGP in the case of the presence of venous spread (*p = 0.035;* that is, venous spread was significantly more frequent in pushing HGP than in desmoplastic HGP). The Kruskal-Wallis test indicated a significant difference in the largest macroscopic diameter of the liver metastasis across HGP groups (*p = 0.023*). Post-hoc pairwise comparisons were performed using the DSCF test with control for multiple testing, and a significant difference was observed between replacement HGP and desmoplastic HGP (*p = 0.037*; that is, the largest macroscopic diameter was significantly larger in patients with replacement HGP than those with desmoplastic HGP). The examined clinicopathological parameters adjusted to HGPs, and the results of statistical analysis are summarised in Table 1.

No significant association was found either with age (*p = 0.284*), gender (*p = 0.898*), the largest diameter of primary tumour (*p = 0.845*), clinical stage (*p = 0.836*), the T, N, or M categories (*p = 0.634; p = 0.087; p = 0.601*), or complete resection (*p = 0.933*). Secondary tumour focality (*p = 0.967*) was not found to be significantly associated with HGPs, either.

Furthermore, while the majority of cases comprised colorectal carcinoma metastasis, another set of statistical analyses was performed. A significant association was found between HGPs and both N stage and the largest metastasis diameter in colorectal carcinoma cases. Concerning N stage, the overall association was also significant (*p = 0.014*), with pairwise differences observed between the replacement and pushing HGPs (*p = 0.032*), and between the desmoplastic and pushing HGPs (*p = 0.006*). The metastasis diameter differed significantly across HGP groups (*p = 0.004*), with significantly greater largest macroscopic diameter in patients with replacement HGP compared to those with desmoplastic HGP (*p = 0.008*). The results of colorectal carcinoma variables are listed in Table 2.

**TABLE 2:** Clinicopathological parameters of colorectal carcinoma cases and results of statistical analysis.

Variables	All cases (n = 163)	Replacement HGP (=78)	Desmoplastic HGP (n = 68)	Pushing HGP (n = 17)	p values
**Mean age (years) [standard deviation]**	68.1 [9.2]	70.3 [8.7]	67.8 [9.9]	67.1 [13.4]	*0.256*
**Gender**					*0.985*
Male	98 (60.1%)	47 (60.2%)	41 (60.3%)	10 (58.8%)	
Female	65 (39.9%)	30 (39.8%)	27 (39.7%)	7 (41.2%)	
**Histological subtypes**	163 (100%)				*1*
Colon adenocarcinoma	102 (62.6%)	49 (62.8%)	42 (61.7%)	11 (64.6%)	
Colon mucinous adenocarcinoma	5 (3%)	2 (2.6%)	1 (1.5%)	2 (11.8%)	
Rectum adenocarcinoma	52 (31.9%)	25 (32%)	23 (33.8%)	4 (23.6%)	
Rectum mucinous adenocarcinoma	4 (2.5%)	2 (2.6%)	2 (3%)	0 (0%)	
**Median of largest diameter of primary tumour (mm) [range]**	32 [4–142]	35 [10–95]	32 [5–84]	35.5 [4–142]	*0.845*
**Clinical stage at the time of primary tumour diagnosis**					*0.639*
Stage 1	9 (5.5%)	2 (2.6%)	5 (7.4%)	2 (11.8%)	
Stage 2	38 (23.3%)	19 (24.4%)	15 (22.2%)	4 (23.6%)	
Stage 3	85 (52.1%)	43 (55.1%)	34 (50%)	8 (47.2%)	
Stage 4	31 (19.1%)	14 (17.9%)	14 (20.4%)	3 (17.4%)	
Pathological stage at the time of primary tumour diagnosis					
T stage					*0.658*
T1	2 (2.9%)	0 (0%)	2 (2.9%)	0 (0%)	
T2	16 (9.8%)	5 (6.4%)	9 (13.2%)	2 (11.8%)	
T3	119 (73%)	60 (76.9%)	45 (66.2%)	14 (82.3%)	
T4	26 (14.3%)	13 (16.7%)	12 (17.7%)	1 (5.9%)	
N stage					***0.014***
N0	65 (39.9%)	34 (46.8%)	22 (32.4%)	9 (52.8%)	
N1	72 (44.2%)	33 (42.3%)	37 (54.4%)	2 (11.8%)	
N2	26 (15.9%)	11 (14.1%)	9 (13.2%)	6 (35.4%)	
M stage					*0.940*
M0	135 (82.8%)	65 (83.4%)	56 (82.4%)	14 (82.4%)	
M1	28 (17.2%)	13 (16.6%)	12 (17.6%)	3 (17.6%)	
**Grade of primary tumour**					*0.962*
Grade 1	4 (2.5%)	2 (2.6%)	2 (2.9%)	0 (0%)	
Grade 2	153 (83.8%)	72 (92.2%)	64 (94.2%)	17 (100%)	
Grade 3	6 (3.7%)	4 (5.2%)	2 (2.9%)	0 (0%)	
**Presence of venous spread in primary tumour specimen**					*0.3*
Present	26 (15.9%)	16 (12.8%)	7 (10.3%)	3 (17.6%)	
Not present	137 (84.1%)	62 (87.2%)	61 (89.7%)	14 (82.4%)	
**Resection of primary tumour**					*0.428*
Complete	158 (96.9%)	74 (94.9%)	66 (97%)	17 (100%)	
Incomplete	5 (3.1%)	3 (5.1%)	2 (3%)	0 (0%)	
**Focality of metastasis**					*0.916*
Unifocal	88 (53.9%)	41 (52.6%)	37 (54.4%)	10 (58.8%)	
Multifocal	75 (46.1%)	37 (47.4%)	31 (45.6%)	7 (41.2%)	
**Median of largest diameter of liver metastasis (mm) [range]**	30 [4–115]	35 [6–97]	24.5 [4–115]	41 [6–78]	***0.004***

Bold p values indicate statistical significance (p<0.05).

Non-colorectal carcinoma cases were also investigated separately. A significant association was found between HGPs and several clinicopathological features. Major histological subtype showed a significant association (*p = 0.009*), with a significant difference between replacement and pushing HGPs (*p = 0.006*). Moreover, tumour grade was significantly associated with HGPs (*p = 0.014*): a significant difference was detected between patients with replacement HGP and those with pushing HGP (*p = 0.034*). Furthermore, a significant association was observed between HGP groups and the presence of venous spread (*p = 0.018*), with a significant difference between desmoplastic and pushing HGPs (*p = 0.046*). The results of this cohort are summarised in Table 3.

**TABLE 3:** Clinicopathological parameters of non-colorectal carcinoma cases and results of statistical analysis.

Variables	All cases (n = 42)	Replacement HGP (=21)	Desmoplastic HGP (n = 9)	Pushing HGP (n = 12)	p values
**Mean age (years) [standard deviation]**	65.2 [10.5]	65.9 [9.9]	62 [15]	66.5 [8.5]	*0.732*
**Gender**					*0.652*
Male	17 (40.5%)	7 (33.3%)	4 (44.4%)	6 (50%)	
Female	25 (59.5%)	14 (66.7%)	5 (55.6%)	6 (50%)	
**Histological subtype of primary tumours**	42 (100%)				***0.009***
Extrahepatic biliary duct carcinoma	4 (9.5%)	4 (19%)	0 (0%)	0 (0%)	
Gallbladder carcinoma	4 (9.5%)	3 (14.4%)	1 (11.1%)	0 (0%)	
Gastric adenocarcinoma	4 (9.5%)	2 (9.6%)	0 (0%)	2 (16.7%)	
No special type carcinoma of the breast	4 (9.5%)	2 (9.6%)	1 (11.1%)	1 (8.3%)	
Pancreatic ductal adenocarcinoma	3 (7.1%)	2 (9.6%)	1 (11.1%)	0 (0%)	
Malignant melanoma	3 (7.1%)	2 (9.6%)	0 (0%)	1 (8.3%)	
Intestinal NET	3 (7.1%)	0 (0%)	1 (11.1%)	2 (16.7%)	
Intestinal NEC	2 (4.7%)	0 (0%)	0 (0%)	2 (16.7%)	
Pancreatic NET	2 (4.7%)	0 (0%)	0 (0%)	2 (16.7%)	
Cervical squamous cell carcinoma	1 (2.4%)	1 (4.7%)	0 (0%)	0 (0%)	
Pulmonary basaloid squamous cell carcinoma	1 (2.4%)	1 (4.7%)	0 (0%)	0 (0%)	
Mesopharyngeal squamous cell carcinoma	1 (2.4%)	1 (4.7%)	0 (0%)	0 (0%)	
Urothelial carcinoma	1 (2.4%)	1 (4.7%)	0 (0%)	0 (0%)	
Gastric NET	1 (2.4%)	1 (4.7%)	0 (0%)	0 (0%)	
Colon leiomyosarcoma	1 (2.4%)	1 (4.7%)	0 (0%)	0 (0%)	
Hypopharyngeal squamous cell carcinoma	1 (2.4%)	0 (0%)	1 (11.1%)	0 (0%)	
Papillary renal cell carcinoma	1 (2.4%)	0 (0%)	1 (11.1%)	0 (0%)	
TFE3 translocation renal cell carcinoma	1 (2.4%)	0 (0%)	1 (11.1%)	0 (0%)	
Mixed germ cell tumour^a^: 1	1 (2.4%)	0 (0%)	1 (11.1%)	0 (0%)	
Pulmonary small cell carcinoma	1 (2.4%)	0 (0%)	1 (11.1%)	0 (0%)	
Prostatic adenocarcinoma	1 (2.4%)	0 (0%)	0 (0%)	1 (8.3%)	
Gallbladder NET	1 (2.4%)	0 (0%)	0 (0%)	1 (8.3%)	
**Median of largest diameter of primary tumour (mm) [range]**	29 [5–95]	45 [5–57]	30 [10–95]	33 [19–48]	*0.338*
**Clinical stage at the time of primary tumour diagnosis**					*0.284*
Stage 1	7 (16.6%)	5 (23.8%)	1 (11.1%)	1 (8.3%)	
Stage 2	9 (21.4%)	8 (38.2%)	0 (0%)	1 (8.3%)	
Stage 3	13 (31%)	4 (19%)	3 (33.3%)	6 (50.1%)	
Stage 4	13 (31%)	4 (19%)	5 (55.6%)	4 (33.3%)	
Pathological stage at the time of primary tumour diagnosis					
T stage					*0.848*
T1	6 (14.3%)	2 (9.5%)	2 (22.2%)	2 (16.6%)	
T2	21 (50%)	11 (52.5%)	4 (44.5%)	6 (50.1%)	
T3	10 (23.8%)	6 (28.5%)	1 (11.1%)	3 (25%)	
T4	5 (11.9%)	2 (9.5%)	2 (22.2%)	1 (8.3%)	
N stage					*0.148*
N0	23 (54.8%)	14 (66.7%)	5 (55.6%)	4 (33.2%)	
N1	10 (23.8%)	2 (9.5%)	2 (22.2%)	6 (50.2%)	
N2	9 (21.4%)	5 (23.8%)	2 (22.2%)	2 (16.6%)	
M stage					*0.199*
M0	32 (76.2%)	18 (85.7%)	5 (55.6%)	9 (75%)	
M1	10 (23.8%)	3 (14.3%)	4 (44.4%)	3 (25%)	
**Grade of primary tumour**					***0.014***
Grade 1	11 (16.7%)	2 (9.5%)	1 (11.1%)	8 (66.8%)	
Grade 2	24 (57.1%)	15 (71.5%)	7 (78.8%)	2 (16.6%)	
Grade 3	7 (26.2%)	4 (19%)	1 (11.1%)	2 (16.6%)	
**Presence of venous spread in primary tumour specimen**					***0.018***
Present	10 (23.8%)	4 (19%)	0 (0%)	6 (50%)	
Not present	32 (76.2%)	17 (81%)	9 (100%)	6 (50%)	
**Resection of primary tumour**					*0.216*
Complete	34 (80.9%)	18 (85.7%)	6 (66.7%)	10 (83.4%)	
Incomplete	8 (19.1%)	3 (14.3%)	3 (33.3%)	2 (16.6%)	
**Focality of metastasis**					*0.718*
Unifocal	23 (54.8%)	13 (61.9%)	4 (44.4%)	6 (50%)	
Multifocal	19 (45.2%)	8 (38.1%)	5 (55.6%)	6 (50%)	
**Median of largest diameter of liver metastasis (mm) [range]**	20 [5–149]	20 [6–135]	18 [5–44]	27.5 [9–149]	*0.545*

Abbreviations: NEC, Neuroendocrine carcinoma; NET, Neuroendocrine tumour, TFE3 - Transcription factor E3.

^a^ 50% yolk sac, 50% postpubertal teratoma. Bold p values indicate statistical significance (p<0.05).

## Discussion

The relationship between the tumourous and non-tumourous liver parenchyma, therefore, the predecessor of HGPs, was reported first by Nakashima et al in 1982. The study comprised 60 cases of hepatocellular carcinoma, and HGPs were identified as sinusoidal, replacing, and encapsulated. The authors stated that HGPs indicate tumour behaviour, while the replacing type cases showed worse prognosis, due to their spread in a rather expansive manner. Those cases with sinusoidal spread reflected aggressive spread since discohesive tumour cells tend to invade more easily [11].

The study of Terayama et al from 1996 consisted of 100 autopsy cases of liver metastases, originating mainly from the lung, pancreas, stomach, gallbladder, bile ducts, and colon; therefore, this publication could be counted as the first one that consecutively examined HGPs. They first macroscopically classified the cases, then they compared the portal type HGP to lymphangitis carcinomatosa of lung cancer, stating that if the tumour cells invade the lymphatic vessels of the portal tract, the peripheral liver spread can occur more effortlessly. The authors also stated that regardless of the histological subtype of the primary tumour, liver metastases tend to first grow in a replacement, and/or sinusoidal manner, and later it would transform to the sinusoidal form, and behave more aggressively. Their results reflect the general knowledge that cellular adhesion would result in expansive tumour growth, while discohesive tumour cells would rather grow in a replacement manner. The prognostic value of HGPs was not examined in this study [16].

The focus shifted to colorectal cancer metastases in 2001, because of the promising results of Vermeulen et al in 2001. The authors identified 3 patterns, namely, replacement, desmoplastic, and pushing. The replacement pattern was associated with unpreserved liver parenchyma, indicated by the loss of reticulin staining, and altered angiogenesis, due to the loss of cluster of differentiation 34 (CD34) of endothelial cells and alpha-smooth muscle actin (SMA) mural cells, while these were preserved in the desmoplastic and pushing patterns. Apoptosis of tumour cells was associated with pushing subtype [2]. Based on these results, the idea that HGPs could be the indicators of the effect of anti-vascular endothelial growth factor (VEGF) seems plausible [10].

The first non-epithelial HGP study was published by Grossniklaus et al in 2016, and 15 uveal melanoma metastases were examined, and so-called infiltrative and nodular patterns were identified. The infiltrative pattern has been associated with sinusoidal space infiltration, while the nodular pattern has corresponded with angiogenesis [17].

The first international consensus guidelines for the evaluation of HGPs were published by van Dam et al in 2017 and were based on 24 studies, including both case reports and original research articles. The identified HGPs were replacement, desmoplastic, pushing, sinusoidal and portal, the latter 2 being rarer subtypes. Twelve participants evaluated 159 cases of colorectal and breast cancer liver metastases, and good-to-excellent agreement was reached with the intraclass correlation (intraclass correlation coefficient: >0.5), and a significant difference was observed between the desmoplastic and replacement subtypes in OS (*p = 0.006*) [18].

An updated guideline and the largest literature review so far have been initiated by Latacz et al in 2022 [13]. The paper divides the existing literature based on methodology. Animal models were used in 7 publications, while 5 studies focused on the immunological background, with the use of immunohistochemical markers, comprising mainly colorectal carcinomas; however, other types of gastrointestinal tumours, including breast carcinomas, and melanomas were included, as well. HGP scoring was examined in 3 studies, in colorectal and pancreatic adenocarcinoma cases, and melanomas. The correlation with imaging techniques was also examined in 6 publications. Regarding the evaluation of HGPs, 2 main methods were defined. One of them is based on choosing a predominant pattern (n = 3), while the other is based on 100% desmoplastic morphology, or with any percentage of replacement pattern (n = 10). However, several articles (n = 8) were not based on any guidelines. Tumour biology was examined by 12 papers, with immunohistochemistry, immunofluorescence, and molecular diagnostics. By that time, 16 reviews were published [13].

Many recent papers indicate that HGPs should be categorised based on whether they contain a desmoplastic pattern at all, or not, while it has been associated with better outcomes [19]. Furthermore, desmoplastic HGP has been linked to the effectiveness of preoperative chemotherapy, as well [20]. The desmoplastic pattern has been associated with a higher CD8+/CD4+ ratio, compared to cases with no desmoplastic pattern [21, 22]. In a recent study involving an animal liver cancer model, replacement type HGP has been linked to metastasis formation, and further supported the above-mentioned data, while an association was found between desmoplastic HGP and hypoxia-inducible factor 1, alpha subunit (HIF1A) and VEGF [23]. Replacement pattern was associated with Claudin 2, therefore, with tumour dissemination, and early cancer cell survival [24]. Through transcriptomics, a novel study proved that replacement HGP is related to the overexpression of genes involved in the cell cycle, DNA repair, and cell motility, whilst desmoplastic HGP is associated with angiogenesis and several immune processes [25].

It has to be emphasised that there have been discrepancies regarding the interpretation of OS, while some authors have performed statistical analysis of HGPs and POOS, metastasis-specific and metastasis-free OS, and others defined 5-year OS in their studies [5, 7, 19, 26–29]. Furthermore, in some papers, OS is not defined at all [30].

Additionally, despite the amount of literature data, HGPs were not extensively studied in a consecutive setting. Meyer et al’s study from 2022 comprised non-colorectal, non-neuroendocrine tumours, including altogether 132 cases of oesophageal, gastric, small intestinal, anal, pancreatic, ampullary, adrenocortical, renal, cervical, endometrial, ovarian, urothelial, breast, otolaryngeal, thymic, and non-small cell lung carcinomas, nephroblastoma, gastrointestinal stromal tumour, leiomyosarcoma, liposarcoma, malignant melanoma, non-seminomatous germ cell tumours, and hemangiopericytoma. Based on their results, a significant association was found between HGPs, RFS, and OS; however, clinicopathological parameters were not examined [9].

In a similar study from Bohlok et al from 2023, 263 cases, including oesophageal, small bowel, colorectal, anal, pancreatic, renal, ovarian, breast, otolaryngeal carcinomas, gastrointestinal stromal tumour, metastatic leiomyoma, malignant melanoma, hemangiopericytoma, and testicular tumours, without indication of seminomatous or non-seminomatous origin, a significant association was found between HGPs, POOS, and PFS; however, clinicopathological parameters were not examined in association with HGPs [5].

In our retrospective, consecutive, single-center study, altogether 336 liver metastases from 205 patients have been included. Most examined cases were of colorectal origin (n = 164), but mesenchymal, melanocytic, and germ cell tumours were also incorporated, as well. A significant association was found between HGPs and histological subtype (*p < 0.001*), grade (*p = 0.002*), the presence of venous spread (*p = 0.02*), and the largest macroscopic diameter of liver metastasis (*p = 0.023*), respectively. However, this study may be limited by its consecutive nature, while different tumour subtypes in such diverse proportions indicate differing biological behaviour and might influence outcomes.

While the examination of HGPs in a consecutive setting is still insufficient, it is challenging to compare our results with the existing ones. The evaluation of clinicopathological factors, including the above-mentioned, significantly associated factors, was not examined in either Meyer’s or Bohlok’s papers; therefore, this could be considered a major advantage for our paper. Furthermore, clinicopathological factors, such as grade, the presence of venous spread, and the largest macroscopic diameter of the liver metastasis, were not yet associated with HGPs in consecutive settings. Controversies still remain regarding the prognostic utility of HGPs, and their examination in a consecutive setting remains in great need of further investigation.

## Data Availability

The raw data supporting the conclusions of this article will be made available by the authors, without undue reservation.

## Abbreviations

CD34, Cluster of differentiation 34; DNA, Deoxyribonucleic acid; DSCF, Dwass-Steel-Critchlow-Fligner; HE, Hematoxylin and eosin; HGP, Histopathological growth pattern; HIF1A, Hypoxia-inducible factor 1, alpha subunit; NA, Not applicable; NEC, Neuroendocrine carcinoma; NET, Neuroendocrine tumour; NOS, Not otherwise specified; NST, No special type carcinoma; OS, Overall survival; PFS, Progression-free survival; POOS, Post-operative overall survival; RFS, Recurrence-free survival; SMA, Smooth muscle actin; TFE3, Transcription factor E3; VEGF, Vascular endothelial growth factor.
